# The (Spatial) Memory Game: Testing the Relationship Between Spatial Language, Object Knowledge, and Spatial Cognition

**DOI:** 10.3791/56495

**Published:** 2018-02-19

**Authors:** Harmen B. Gudde, Debra Griffiths, Kenny R. Coventry

**Affiliations:** ^1^School of Psychology, University of East Anglia

**Keywords:** Behavior, Issue 132, Memory game, Spatial Cognition, Object knowledge, Spatial Language, Spatial Memory, peri/extrapersonal space, Cross-linguistics

## Abstract

The memory game paradigm is a behavioral procedure to explore the relationship between language, spatial memory, and object knowledge. Using two different versions of the paradigm, spatial language use and memory for object location are tested under different, experimentally manipulated conditions. This allows us to tease apart proposed models explaining the influence of object knowledge on spatial language (*e.g.*, spatial demonstratives), and spatial memory, as well as understanding the parameters that affect demonstrative choice and spatial memory more broadly. Key to the development of the method was the need to collect data on language use (*e.g*., spatial demonstratives: "*this/that"*) and spatial memory data under strictly controlled conditions, while retaining a degree of ecological validity. The language version (section 3.1) of the memory game tests how conditions affect language use. Participants refer verbally to objects placed at different locations (*e.g.*, using spatial demonstratives: "*this/that* red circle"). Different parameters can be experimentally manipulated: the distance from the participant, the position of a conspecific, and for example whether the participant owns, knows, or sees the object while referring to it. The same parameters can be manipulated in the memory version of the memory game (section 3.2). This version tests the effects of the different conditions on object-location memory. Following object placement, participants get 10 seconds to memorize the object's location. After the object and location cues are removed, participants verbally direct the experimenter to move a stick to indicate where the object was. The difference between the memorized and the actual location shows the direction and strength of the memory error, allowing comparisons between the influences of the respective parameters.

**Figure:**
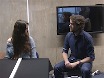


## Introduction

The relationship between language and non-linguistic representations is a fundamental topic in the cognitive sciences^1^,^2^,^3^,^4^. In exploring this relationship, we focus on spatial cognition. The memory game procedure enables us to experimentally control the influence of different parameters on the relations between spatial language, spatial memory, and object knowledge, while also retaining a degree of ecological validity. Past methods used to elicit spatial demonstratives or the comprehension of them range from those that have high ecological validity but low experimental control (*e.g.*, the observational work of Enfield^5^, or the elicitation methods developed in Max Planck Institute field guides^6^) to those that have high experimental control but low ecological validity (such as the within-participant designs employed tapping the congruence of demonstratives with pictures^7^,^8^). The memory game method was developed not as a substitute for these methods, but rather as a complementary method retaining the strengths of these various approaches within a single paradigm.

Key to the development of the method was the desire to retain high experimental validity while also ensuring that participants use language naturalistically in (real) three-dimensional space without being aware that their language was being tested. There are several important points to note here. First, the brain systems underlying peri-personal (near) space and extra-personal (far) space in vision and action are reasonably well charted, and involve a (graded) distinction between the space reachable around the body and the space not reachable^9^,^10^,^11^. In past linguistic investigations of the influence of distance on demonstratives, the perceptual basis of this distance distinction is often not adequately considered. The use of photographs in some past studies manipulating distance where the whole image on the screen is in peripersonal space is arguably not a fair test of the influence of distance on demonstratives as motivated from the basic brain system distinction. Second, asking participants to produce demonstratives and telling them that the researchers are interested in the demonstratives used opens up the possibility of bias, with participants generating their own theories regarding demonstratives and thus not producing them naturally. For that reason, the memory game uses a cover story to elicit demonstratives without participants realizing that the demonstratives chosen are of interest. Indeed, on debriefing we find that participants report being unaware of the real purpose of the study. Moreover when the purpose of the study is revealed, participants often describe how they use demonstratives in ways that do not necessary accord with their own actual behavior during the task.

There are two basic versions of the memory game, exploring language use (from here the 'language version') and object location memory (from here the 'memory version'), in which we can manipulate different parameters (see section 3.3). In the context of questioning research findings exploring top-down effects of cognition on perception^12^, the memory game aims to avoid the pitfalls identified by Firestone and Scholl, such as overly confirmatory research designs (different models are tested, allowing disconfimation) and demand and response biases (cover stories ensure participants are unaware of the aim of the study). (See attachment for a transcript of the instruction for both versions of the memory game.)

In the language version (section 3.1) of the memory game, testing *spatial language production, *we use memory as a cover story so that demonstratives can be elicited without participants realizing that their use is being measured. Participants are instructed that they are taking part in an experiment examining the influence of language on memory for object location (the experiment is advertised as a memory experiment). Participants sit at a long table with a number of color-coded locations marked at various distances in front of them. At the start of each individual trial, the experimenter or the participant (*agent* is a potential experimentally controlled parameter) places an object (*e.g.*, blue heart, black cross) at one of the locations. Between trials, the distance from a participant is varied, as well as other parameters of potential interest, such as ownership (whether the participant owns an object or not), visibility, familiarity, agent (who places the object), and the position of a conspecific. After object placement, participants point at the object (but do not touch it) and name it. Participants are instructed to use three words: demonstrative, color, object name (*e.g.*, for the English version: "*this/that* red circle") .

**Figure:**
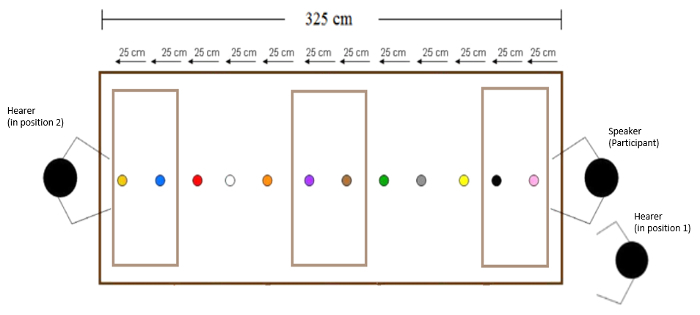

Figure 1. Overview of the table setup and positions of speaker (participant) and hearer (experimenter). Adjusted from Coventry et al.**1**Please click here to view a larger version of this figure.

**Figure:**
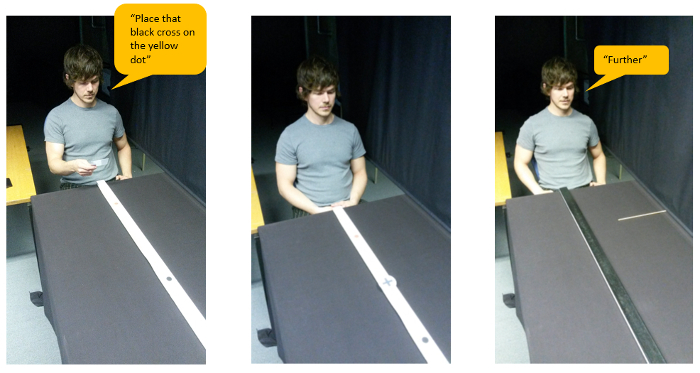

Figure 2. The participant reads out the instruction card, then memorizes the object location and finally instructs the experimenter to move the indication stick and align it with where the edge of the object was**14**. Please click here to view a larger version of this figure.

In the second version of the memory game (section 3.2), we test *memory for object location*. Following object placement, participants view the object/location for 10 seconds. After the 10 seconds, the object and location markers are removed, and participants verbally instruct the movement of an indication stick (see Figure 3, the indication stick is placed at various distances, either closer or farther from the actual location) to match the exact location they thought the near edge of the object was at. The experimenter moving the indication stick stands behind a curtain to avoid a clever Hans effect (*i.e., *so that the participant cannot read any accuracy clues from the experimenters' face/body language). The difference between the recalled location and the actual object location shows the direction and strength of the influence of the respective conditions (see Figure 4).

Across multiple series of experiments employing these procedures, we found a close relation between spatial language and spatial memory [*e.g*., see **Table 1**]. Distance and multiple parameters of object knowledge (*e.g.*, ownership, visibility, familiarity) have been found to influence the use of demonstratives (*this/that)*^13^. For example, if an object is out of reach, one is more likely to say *that* compared to *this*^1^,^13^; if the object placed is owned by the participant, one is more likely to use *this *compared to when the placed object is owned by someone else. Furthermore, results from the memory version parallel results from the language production version. In situations in which participants are more likely to refer to an object using *that *compared to *this*, participants misremember the object to be further away in the memory version^13^.This effect is also extended to language at instruction affecting spatial memory: if the object is placed with the word *that *instead of *this* (*e.g.*, if the participant reads out an instruction for object placement: "Place *that *object on the location"), participants misremember the object to be further away in the memory version^14^. More specifically, the influence that object knowledge has on spatial language and memory (*e.g.*, objects placed further away are verbalized using the demonstrative *that *rather than* this) *is similar to the influence that spatial language has on spatial memory (objects placed with *that* are misremembered to be further away than placed with *this*). This shows a close relation between spatial cognition and language.

Theoretically these methods have been used to differentiate between several possible models predicting different influences of spatial language on spatial memory. For example, the Expectation model^13^ suggests that spatial memory is a concatenation of the expected location and the actual location. For instance, if one owns an object, it is expected that the object will be closer than if the objects belongs to someone else (since most objects that are close by are objects one owns). The expectation model is based on previous results^1^,^13^, and the evidence across experiments favors this model over other models in follow-up studies^14^.

## Protocol

All methods described here have been approved by the School of Psychology Ethics Committee of the University of East Anglia.

### 1. Preparation

1. Advertise the experiment as a memory experiment; it is important for the language production version that language is never mentioned as the dependent variable.
2. Inform all participants in accordance with ethical guidelines, and let them sign a consent form confirming they agree to take part under the following presumptions:
  1. Presume that participants understand the information provided.
  2. Presume that participants had the chance to ask questions about the experiment and had these answered satisfactorily.
  3. Presume that participants know their participation is voluntary and that they are allowed to withdraw at any time, without giving any reason and without it affecting them in any way.
  4. Presume that participants know that no personal information (such as a name) will be shared outside of the research team or published in the final report(s) from the research.
3. Materials
  1. Cover a standard height table (L = 325 cm, W = 90 cm) with a single-colored tablecloth to prevent any marks on the table from being a spatial marker for participants. The length of the table may be varied as a function of the distances to be tested.
  2. Mark locations, spaced equidistantly on a location-stick, placed along a midline from the participant (who sits at a short end of the table, See Figure 1). Place the objects on the near edge of the location marks, so that there is no variation in the exact location of the placed objects. NOTE: The number of marked locations and their distances may be varied as a function of the tested parameters. In the video we use 12 locations, with a distance of 25 cm between them.
  3. Print different colored shapes and place them in plastic discs (6.5 cm in diameter), thus controlling for object properties. Other objects can be used, dependent on the hypotheses to be tested; for example in one experiment manipulating ownership coins owned by either the participant or experimenter were used (See Figure 5).
  4. Cover the space around the table with curtains, in order to eliminate any distance cue from the room.
  5. Cover one of the long sides of the table with a curtain [see Figure 4]. In the memory version (section 3.2) of the procedure, the experimenter is behind this curtain while the participant indicates where s/he remembers that the object was. This ensures the participant cannot see the experimenter, preventing any non-verbal cues as to the accuracy of the participants' response. In turn, the experimenter cannot see the table, thus eliminating any confounds in moving the indication stick.
  6. Place a measuring tape on the table on the experimenters' side of the curtain, out of sight of the participants, in order to record the recalled location in the memory version (section 3.2).
  7. To avoid being influenced by the tape measure during the participants' instructions, have the experimenter focus only on moving the stick at a constant speed. This way, the experimenter does not have information on accuracy while moving the indication stick. When the participant states that the near edge of the indication stick matches the near end of the object, use the measuring tape to note the recalled location of the object.
4. Participants
  1. Make sure participants are native speakers of the tested language, and (ideally) do not speak another native language. (Of course, the memory game can be used for bilingual studies/testing other populations if desired.)
  2. Ensure participants have a depth perception of at least 40" (arcseconds), which can be measured with depth perception tests (*e.g*., Randot Stereopsis test; see table of materials).

### 2. Procedure

NOTE: Some details of the setup depicted in the video differ slightly from the manuscript. For example, the location—stick looks somewhat off centre (especially when turned over), the position of the experimenter next to the participant, and the lighting conditions (resulting in shadows), were adjusted for videographic reasons. This should be avoided during testing, to avoid confounding visual cues. Follow the details in paper when replicating.

1. Seat the participant as close as comfortable to the short side of the table (but without actually touching it).
2. Instruct the participant to try and maintain their position throughout the experiment.
3. Tell the participant which objects and which locations will be used during the experiment.
4. Present the participant with six practice trials, using the 6 different objects and locations, so that any uncertainties about the procedure are noticed and explained.
5. Instruct participants following the instruction transcript (Supplementary File).
6. Adjust the procedure to specific testing needs. For example, different parameters can be manipulated or fewer trials can be used to keep to a specific timeframe (see section 3.3).
7. At the end of the task, ask participants what they thought of the experiment, and specicially what they thought the experiment was testing. This is particularly important for the language version where participants need to be eliminated from the analyses should they report that their demonstrative choice was being tested.

### 3. Variants

1. **Language version** NOTE: The language version of the memory game assesses how spatial language is used to verbalize spatial situations.
  1. First instruct participants that they are taking part in an experiment testing the influence of language on spatial memory (so that participants do not realize that their demonstrative use is being measured). Tell participants that the experiment is testing the influence of language on memory for object location, and that they are taking part in the 'language condition' (with other people - not actually tested - in the 'no language' condition). Instruct participants to use three words: [a demonstrative], [the color of the object], [the shape of the object] (*e.g.*, *this/that* black cross), in order to make this as similar as possible for all participants.
  2. On each trial, the experimenter will instruct the placement of an object (*e.g.*, "I/ You place the [object] on the [location]"). The agent (who places) condition is one of the experimentally controlled parameters.
  3. Present an example trial in which the experimenter places the object and shows what a trial looks like, for example: "I place the [object] on the [location]."
  4. Create multiple trial lists, randomizing the order of trials while ensuring no object or location is used in two successive trials. This prevents trial order effects and carry-over effects from trial to trial.
  5. After the object placement, when both experimenter and participant are seated, have the participant use body language (only to point at the object, but not touch it) and verbal language to name the object.
  6. Instruct the participant to name the object, only using three words: [demonstrative], [object color], [object name] (e.g., "this blue heart"). NOTE: The participant does not name the location. Participants are informed that they must use only three words so that the 'language condition' participants are all using the same amount of language to potentially affect memory.
  7. As an experimenter, record which demonstrative term the participant uses to refer to the object. (From the partcipant's perspective, it appears that the experimenter is marking off completed trials.)
  8. After the participant names the object, remove the object and proceed with the instruction for the next trial.
  9. Maintain the 'memory game' cover of the language version of the experiment, by asking the participant to recall the most recent locations of four of the objects, at six or more different times, presented throughout the experiment (so at least 24 memory trials in total; this can be varied as a function of the total number of trials).
2. **Memory version** NOTE: The memory version assesses how manipulated conditions (*e.g.*, ownership, language, see section 3.3), affect memory for object location.
  1. Instruct the participant to read out an instruction card at the start of every trial, instructing the placement of an object. The instruction card allows the manipulation of a language condition if desired.
  2. During object placement, ensure participants have their eyes closed. Following object placement, control for memory encoding time and ensure participants get exactly 10 seconds to encode object location memory (look at the object).
  3. After the 10 seconds of memory encoding, instruct participants to close their eyes and turn the location-stick turned upside down so that participants cannot see the locations during the memory recall [see Figure 4].
  4. Make sure both experimenter and participant are at their respective places during the recall (see Figure 1). In this setup, a second experimenter will move the indication stick, out of sight of the participant.
  5. Place the indication stick at varying distances from the actual location (counterbalanced to be closer by or further from the participant), so that participants have to instruct the movement of the indication stick to reach the actual location.
  6. To prevent the placement of the indication stick becoming an anchor for participants, within the first 10 trials, add 3 filler trials. At these filler trials, place the indication stick at more extreme distances (e.g., 20 cm) from the actual location. These filler trials are not used in the analysis.
  7. Instruct the participant to verbally indicate whether the indication stick needs to be 'further' or 'closer' to match the recalled location of the object.
  8. When the participant is satisfied with the location of the indication stick, instruct them to say 'stop.'
  9. Note the recalled location, using the measure tape on the side of the table.
  10. Repeat a trial at the end of the experiment if the participant memorizes an object to be more than 10 cm from the actual location.
3. **Variants on Tasks** NOTE: Variants on these tasks can be used in both the language version (section 3.1) and the memory version (section 3.2).
  1. To manipulate the agent, use instructions that indicate whether the experimenter or the participant places the object.
  2. To manipulate ownership, provide the participant with coins (*e.g*. as part of the participation payment). By using the participants' coins and the experimenters' coins, the ownership variable is manipulated.
  3. Use different object colors and forms to manipulate familiarity (*e.g.*, for familiar: red circle; for unfamiliar: viridian nonagon).
  4. Cover objects during encoding to manipulate visibility. A metal cover for the no-visibility and no-touch condition, a glass cover for the visibility but non-touch condition, or no cover for the visibility and possibility to touch condition. More information about these manipulations can be found in Coventry *et al.* (2014).
  5. To manipulate language at encoding in the memory version, use instruction cards on which language is manipulated (*e.g.*, the participant reads out instruction cards like "Place *this/that/the *[object] on the [location]"). The instruction cards are printed on laminated cards the size of playing cards. Present these cards in a card-shoe, placed on the objects-table, to ensure easy access for the participant.
  6. Repeat a trial when a participant does not remember the demonstrative used on the instruction card at the end of the trial. Ask the participant to recall the demonstrative used on the instruction card at the end of each trial (to ensure participants remember the specific language condition of the trial). To ensure participants do not realize the study is about demonstratives, present this demonstrative recall task as cognitive load task, to make the task more difficult.
  7. To manipulate position of a conspecific, have the conspecific take position either next to the participant, or on the opposite side of the table. In this way, the territory of the participant and experimenter can be shared or positioned opposite. This manipulation is based on linguistic theories suggesting some language (*e.g*., Japanese) require perspective taking when an object is closer to a conspecific. When participant and experimenter are seated opposite one another, locations can be divided by position (*e.g.*, within reach of participant, within reach of experimenter, out of reach for both) and distance from participant (within reach of participant and experimenter, medium far, furthest away). Using this manipulation, a second experimenter needs to move the indication stick on participants’ instruction so that the first experimenter can maintain his/her position.

### 4. Analysis

1. Language version (section 3.1)
  1. Calculate percentages of demonstrative use for each combination of variables.
  2. Analyze data with a mixed ANOVA, using within (*e.g.*, distance, ownership, familiarity, visibility, language at instruction, position of a conspecific) and between (*e.g.*, gender) variables^1^,^13^.
2. Memory version (section 3.2):
  1. Calculate the absolute value of the difference between the actual location and the recalled location (in mm, negative values indicate the object was recalled to be closer) for all trials, then average this difference over each cell of the design.
  2. Analyze data in a mixed ANOVA, using within (*e.g.*, distance, ownership, familiarity, visibility, language at instruction, position of a conspecific) and between (*e.g.*, gender) variables^13^,^14^.

## Representative Results

Results of the 10 experiments to date show a consistent pattern. Demonstrative use is influenced by a number of parameters – distance, agent, ownership, familiarity, visibility, position of a conspecific – whether they are explicitly encoded in a language or not^1^,^13^,^14^ (see Table 1).

Results^1^ suggested that spatial demonstratives are related to areas of peri- and extrapersonal space^15^. These discrete zones of space are flexible can be extended and contracted by tool and weight use^1^,^16^.

Coventry *et al.*^13^ showed experimentally that demonstrative use and memory for object location are related – that is, when participants tend to prefer *that *over *this *under specific conditions (in the language version), participants memorize objects further away in similar conditions (in the memory version) (see Table 2). These language effects are found next to a distance effect (in which the memory error is larger as the object is placed farther away), and a general overestimation of distance from the participant. Results from Gudde *et al.*^14^ showed that objects placed with *that *in the instruction were misremembered to be further away than objects placed with *this* (the same pattern was also found with possessives – *my/your red circle*, etc).

Results support the Expectation model^13^ which proposes that memory for object location is a concatenation of the actual location of an object and the expected location. The expectation of an objects' location can be elicited by general object knowledge (*e.g.*, regarding ownership), or language used to refer to the object (*e.g.*, spatial demonstratives). The expectation model is consistent with theories of predictive coding^17^,^18^. The prediction of other models have been tested against the data (*e.g.*, congruence model, attention attenuation model), but predictions of these respective models do not match the data (see Gudde *et al.*^14^ for discussion).

A second implication is more linguistic in nature. Coventry *et al.*^13^ tested parameters that are explicitly encoded in some languages, but not in English. These parameters were found to influence English demonstrative use, and memory for object location in English participants nonetheless. This fact might suggest that demonstrative systems depend on a universal set of parameters that might reside outside of language (given that spatial memory is affected by the same set of parameters). However, the memory game has only been tested in a few languages to date. Before more languages are tested, it is premature to make claims of universality. Therefore, more experimental testing across a broader range of languages is needed.

**Figure:**
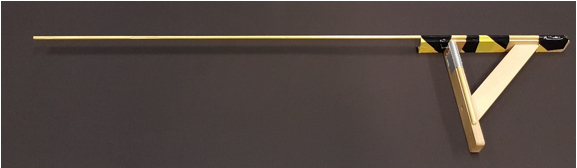

Figure 3. Indication stick**13**.

**Figure:**
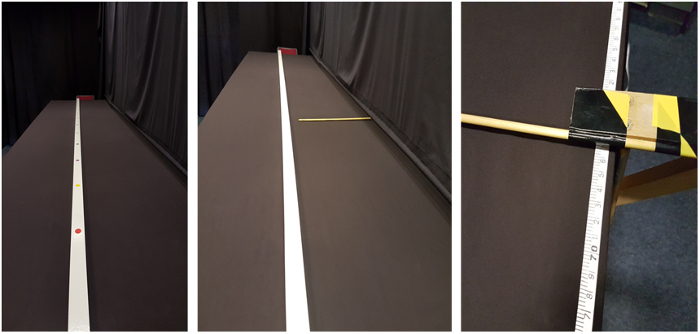

Figure 4. Procedure from left to right: First participants see the locations on the location stick. After the 10 seconds of memory encoding, the locations are taken away and participants are presented with an indication stick. When participants verbally directed the placement of the indication stick, the experimenter notes the actual recalled location using a measuring tape, which is not visible to the participant. Please click here to view a larger version of this figure.

**Figure:**
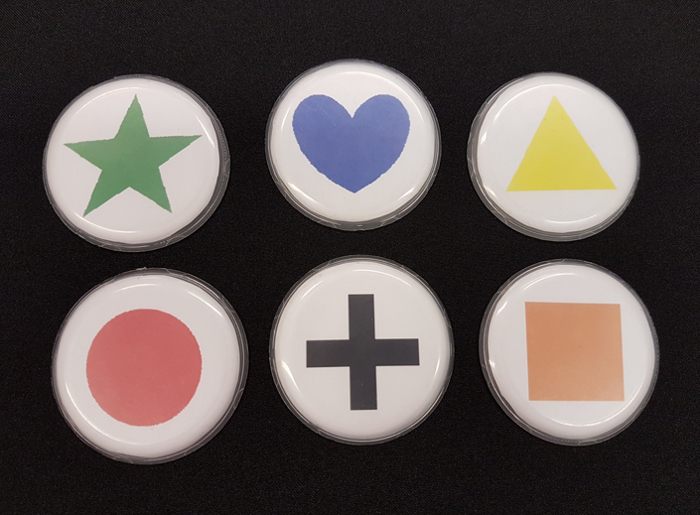

Figure 5. An example of 6 objects. Please click here to view a larger version of this figure.

**Table:** 

**Experiment**	**condition**		**region 1**		**region 2**		**region 3**	
			**(25 - 75 cm)**		**(100 - 150 cm)**		**175 - 225 cm)**	
			**Mean**	**SEM**	**Mean**	**SEM**	**Mean**	**SEM**
**Ownership**	Participant placed	Participant's coins	68.00	6.52	48.00	6.69	18.67	5.80
		Experimenter's coins	62.67	8.01	28.00	6.85	13.33	5.77
	Experimenter placed	Participant's coins	64.00	7.68	29.33	6.75	17.33	5.81
		Experimenter's coins	56.00	7.87	24.00	5.62	9.33	4.52
**Visibility**	Metal (occluded)		41.18	7.84	15.69	7.07	3.92	2.68
	Glass (visible but not touchable)		50.98	8.14	27.45	9.58	5.88	4.27
	No cover (visible and touchable)		60.78	7.69	21.57	6.35	9.80	4.75
**Familiarity**	Familiar shapes		50.76	6.53	39.39	6.06	31.82	5.90
	Unfamiliar shapes		33.33	6.39	28.03	5.94	33.33	6.30

**Table 1.** **Percentage of "this" responses in each condition across the three regions (Coventry *et al*.** **13** **)**

**Table:** 

**Experiment**	**condition**	**25 cm**	**37.5 cm**	**50 cm**	**62.5 cm**	**75 cm**	**87.5 cm**	**100 cm**	**112.5 cm**	**125 cm**
		**Mean**	**Mean**	**Mean**	**Mean**	**Mean**	**Mean**	**Mean**	**Mean**	**Mean**
		**(SEM)**	**(SEM)**	**(SEM)**	**(SEM)**	**(SEM)**	**(SEM)**	**(SEM)**	**(SEM)**	**(SEM)**
**Ownership**	Participant's coins	0.42	0.36	0.76	0.69	1.68	1.50	1.89	2.48	3.38
		(0.54)	(0.45)	(0.58)	(0.6)	(0.49)	(0.7)	(0.69)	(0.72)	(1.15)
	Experimenter's coins	0.35	0.94	2.83	2.60	3.03	3.91	6.74	5.11	6.14
		(0.67)	(0.48)	(0.93)	(0.88)	(0.86)	(0.97)	(2.43)	(0.95)	(1.24)
**Visibility**	Metal (occluded)	2.91	1.96	2.79	3.55	2.17	3.50	3.38	5.28	6.39
		(2.09)	(0.55)	(0.7)	(1.02)	(1.07)	(0.71)	(0.76)	(1.02)	(1.08)
	Glass (visible but not touchable)	1.37	1.67	1.03	0.95	0.43	1.85	1.97	0.61	3.45
		(0.42)	(0.82)	(1.13)	(1.07)	(0.99)	(0.76)	(0.99)	(1.17)	(1)
	No cover (visible and touchable)	-0.09	-0.65	0.52	-0.08	1.40	1.78	2.08	1.35	3.24
		(0.29)	(1.02)	(0.57)	(0.73)	(0.93)	(0.52)	(0.97)	(0.91)	(0.92)
**Familiarity**	Familiar shapes	0.26	-0.24	-0.12	0.03	0.03	0.97	1.62	0.95	2.22
		(0.47)	(0.48)	(0.56)	(0.8)	(0.59)	(1.09)	(1.1)	(1.26)	(0.94)
	Unfamiliar shapes	2.04	2.42	2.70	3.26	5.09	4.88	4.48	4.59	5.49
		(0.44)	(0.76)	(0.69)	(0.83)	(1)	(0.87)	(0.78)	(0.67)	(0.85)

**Table 2. Absolute memory errors (cm) in each condition by distance (Coventry* et al.*** **13** **)**

## Discussion

The memory game has been shown to be an effective procedure to explore the relationship between language, spatial cognition, and object knowledge. The described procedure can be used to test many different parameters. In section 3.3, we provided an overview of parameters tested so far – this is not meant to be an exhaustive list of potential parameters. This publication aims to provide a structure for testing the relationship between language, spatial cognition and object knowledge, but is not meant to restrict conceptual replications. Any step in the procedure can be adjusted if required by the aim of the replication.

The method could easily be adapted to test other types of spatial language. For example, spatial adpositions could be elicited in a language version asking participants to use spatial adpositions to describe object locations (e.g. *next to*, *in front of*, etc.) rather than demonstratives. The table could also be replaced by a vertical plane to examine prepositions on the vertical axes (e.g. *over*, *under*, *above*,* below*; see Coventry and Garrod^3^ for discussion of adpositions more broadly).

The combination of high experimental control and high ecological validity combines the strengths of previous research methods. The lab setting ensures a high level of experimental control, in which specific parameters can be manipulated and contrasted. At the same time, ecological validity is maintained. Participants are generally unaware about the real aims of the study, and use language naturally. The memory game cover means that upon debrief, few participants report the intuition that language was tested. In the language version of the memory game (section 3.1), participants accept that the language is part of an 'encoding' manipulation. In the memory version of the memory game (section 3.2), when language is manipulated at encoding participants accept that the language is used to increase cognitive load.

It is essential to control the exact placement of the objects used, so that the objects are placed at exactly the same locations in all conditions. Other experimental parameters may be altered to suit the relevant research questions. Future research, employing this memory game, can focus on testing the relation between spatial cognition, object knowledge, and languages in cross-linguistic studies.

## Disclosures

The authors have nothing to disclose.
